# Symptom domains and psychosocial functioning in borderline personality disorder

**DOI:** 10.1186/s40479-024-00255-2

**Published:** 2024-06-05

**Authors:** Ines Culina, Setareh Ranjbar, Pauline Maillard, Chantal Martin-Soelch, Sylvie Berney, Stéphane Kolly, Jérémie André, Philippe Conus, Ueli Kramer

**Affiliations:** 1 General Psychiatry Service, Department of Psychiatry, University Hospital Center and University of Lausanne, Lausanne, Switzerland; 2 Unit of Clinical and Health Psychology, Department of Psychology, University of Fribourg, Fribourg, Switzerland; 3 Department of Psychiatry, Psychiatric Epidemiology and Psychopathology Research Center, Lausanne University Hospital and University of Lausanne, Lausanne, Switzerland; 4 Private Practice, Morges, Switzerland; 5 Institute of Psychotherapy, Lausanne University Hospital, University of Lausanne, Lausanne, Switzerland; 6 Departement of Psychology, University of Windsor, Windsor, Canada

**Keywords:** Borderline Personality Disorder, Psychosocial functioning, Symptoms

## Abstract

**Background:**

Borderline personality disorder (BPD) is often characterized by severe functional impairment, even after a decrease in symptoms. A comprehensive understanding of psychosocial functioning in BPD is necessary to tailor treatment offer, which should address relevant aspects of daily life. The aims of the present study are to (1) conduct a cross-sectional comparison of functioning of a group with BPD and a non-BPD clinical comparison group at service entry, and to (2) assess the relationship between intensity of BPD symptom domains and psychosocial functioning.

**Methods:**

The sample consists of *N* = 65 participants with BPD and *N* = 57 participants from the clinical comparison group without BPD (non-BPD group). The Revised Borderline Follow-up Interview (BFI-R) was used to evaluate psychosocial functioning and the Revised Diagnostic Interview for Borderlines (DIB-R) to assess BPD symptoms. Linear, logistic, and multinomial regression models were run separately for each aspect of functioning as a function of BPD status or BPD symptom domains.

**Results:**

Only 23% of participants in the BPD group fulfilled criteria for good overall psychosocial functioning, compared to 53% in the non-BPD group. Furthermore, participants in the BPD group were less likely to have completed a high number of years of education, to work consistently, to be financially independent, to be in a cohabiting relationship and have a good relationship with parents. In addition, various links were identified between BPD symptom domains and functional impairments.

**Conclusions:**

Consistent with prior research, the main impairments in functioning in the BPD group are found in the educational and vocational domains. Though some domains show impairment, others, like friendships, may act as potential resources. Further investigation on the relationships with symptom domains is required.

**Supplementary Information:**

The online version contains supplementary material available at 10.1186/s40479-024-00255-2.

## Background

Individuals with borderline personality disorder (BPD) often face important challenges in their psychosocial functioning, which refers to “a person’s ability to carry out roles and perform activities in daily life, including in social or interpersonal, school or work, recreational or leisure, and basic (i.e., self-care, communication, mobility) functional realms” [1, p.33]. Current evidence shows that individuals with BPD tend to experience important psychosocial impairment [2–4]. Studies assessing functioning in BPD consistently highlight that, despite some heterogeneity, participants with BPD show great impairment in functioning, especially in the vocational domain; moreover, functional impairment tends to persist even after a decrease in symptoms [4–17]. Observations on social and vocational impairment in clinical samples with BPD were supported by observations in community samples [18–22]. These findings are not exclusive to adults, as impairment in psychosocial functioning related to BPD is already observable during adolescence [23–29]; in addition, BPD psychopathology during adolescence is predictive of long term impairment in functioning [19].

Even though functional impairment can result from any mental disorders, research suggests that individuals with BPD are more likely to present difficulties in psychosocial functioning in comparison to almost all other personality disorders [8, 15], as well as mood and anxiety disorders [30]. In terms of vocational outcomes, Hastrup, Kongerslev [31] noted that individuals with BPD displayed worse outcomes than all other psychiatric disorders except for schizophrenia, schizotypal, delusional disorders as well as substance-related disorders.

Moreover, as it may be expected, it has been noted that symptoms significantly impact functioning; specifically, participants with persistent symptoms (never remitted) display worse functioning compared to those who have experienced remission [32]. In this sense, there has been an additional effort in research to further examine the links between symptoms and psychosocial functioning. Within this body of research, mixed results have emerged highlighting various associations with functioning. For instance, these include associations with a chronic feeling of emptiness [33, 34], impulsivity [35], emotion dysregulation [36] and identity disturbance [37]. Of particular interest are studies utilizing the same assessment tools, allowing for direct comparison. For instance, several studies have employed the Revised Diagnostic Interview for Borderlines (DIB-R) [38], of relevance as it is the instrument used in the present study. The DIB-R [38] measures four symptom domains: affects, cognition, impulse action patterns, and interpersonal relationships. To be accurate, the cognition section assesses odd thinking, unusual perceptual experiences, non-delusional paranoid experiences, as well as both true and ‘quasi’ psychotic thoughts. Again, findings vary across studies; the following results of follow-up studies were observed: lower scores in impulsivity domain [39, 40], interpersonal relationships domain [39] and cognitive sector domain [40] were linked to better functioning; moreover, higher scores in affect and impulsivity domains were linked to higher risks of having poor psychosocial functioning [39]. Javaras et al. [20], relying uniquely on a single assessment point, observed that the intensity of all four sector domains was associated to lower odds of good psychosocial functioning; they also showed that different BPD symptom sectors had similar levels of associations with specific functioning outcome. Further studies are needed to better summarize research on the topic and identify more effectively symptomatic predictors of functioning in BPD.

In consideration of the current knowledge on the topic, functional impairment stands out as an enduring and stable aspect of the disorder which acts as the major obstacle to achieving recovery in individuals with BPD and requires further research [6, 34]. In fact, there appears to be effective treatment for reducing BPD symptoms, however, the efficacy of these treatments becomes less conclusive in terms of improving psychosocial functioning, especially when functional improvement is not a specific aim of the therapy. For instance, a meta-analysis exploring the impact of psychotherapy on psychosocial functioning in BPD showed an effect size of 0.41, but with wide confidence intervals [41]. The recent Cochrane review indicated that psychotherapy had positive effects on various outcomes, including psychosocial functioning, however the clinical significance was only achieved specifically for symptoms [42]. In addition, beyond the personal distress experienced by the person and their loved ones, this also results in significant societal costs [43], such as in terms of social security disability insurance [44] or treatment utilization [22, 45]. Due to the afore-mentioned considerations, it is imperative to investigate psychosocial functioning in BPD. This investigation is essential in order to define the characteristics of psychosocial functioning, its predictors, refine therapeutic interventions, and ultimately, offer the possibility of attaining a satisfactory quality of life.

Accordingly, the primary aim of the present study will be to compare psychosocial functioning of a group with BPD and a clinical comparison group of individuals without a diagnosis of BPD. More specifically, the main aspect we will assess is the attainment of good overall functioning, additionally, we will assess specific aspects of functioning within the educational/vocational domain, as well as the interpersonal and social domain. As supported by the existing literature, we hypothesize that the rate of individuals in the BPD group who attain good overall functioning will be significantly inferior to the non-BPD group; we also hypothesize that individuals in the BPD group will display worse functioning in all domains, with greater differences between the groups observed in the educational/vocational domain. An additional secondary aim of the present paper is to investigate the associations between intensity of the four BPD symptom sectors (i.e. affective, cognitive, impulse action pattern, interpersonal domains) and good overall functioning as well as each specific aspect of functioning (within educational/vocational, interpersonal and social domains). To do so, the total sample combining the two groups will be analyzed. Results of previous research on the topic are mixed, for this reason all relationships will be investigated. Still, as reported in previous literature [39, 40, 46], we expect that intensity of the impulse action pattern domain will be associated to lower probability of achieving good overall psychosocial functioning. Relationships between intensity of symptom sectors and specific aspects of functioning are conducted in an exploratory fashion.

## Methods

### Participants

The total sample included *N* = 122 participants recruited in an outpatient unit of a French-speaking University Hospital, with the BPD group comprising *N* = 65 participants and the clinical comparison group of individuals without BPD (non-BPD group) comprising *N* = 57 participants. The total sample had a mean age of 34.4 years (*SD* = 12.1), with *N* = 78 participants (64%) being female. Inclusion criteria for both groups were: being between 18 and 65 years of age and having a sufficient level of French. Exclusion criteria for the total sample were the presence of a psychotic disorder and mental retardation according to the fifth edition of the diagnostic and statistical manual of mental disorders (DSM-5) [47]. Participants were assigned to the BPD subgroup if a DSM-5 diagnosis for BPD was present; otherwise, they were assigned to the non-BPD group. The evaluation of personality disorders was conducted using the structured clinical interview for DSM-5 (SCID-5-CV) [48] and the assessment of additional comorbid disorders was conducted by clinicians. Participants in the non-BPD group underwent a four-session brief intervention, including diagnostic investigation, with the formulation of treatment plans and the recommendation of psychotherapy if necessary, resulting in a variety of diagnosis and problems. Table 1 displays the sociodemographic and diagnostic characteristics of the sample. Expanding on the diagnoses outlined in Table 1, details on the number of diagnosis within each group is of interest for a more nuanced understanding of severity. In the BPD group, 20 participants exclusively received the BPD diagnosis, 14 participants had a total of 2 diagnoses, 15 participants had a total of 3 diagnoses, 12 participants had a total of 4 diagnoses, and 4 participants had 5 or more. In the non-BPD group, 6 participants had no diagnosis at the time of assessment, 42 participants had one diagnosis, 7 participants had two diagnoses, and 2 participants had 3 or more diagnoses.

**Table 1:** Sociodemographic and diagnostic characteristics of the BPD group and the non-BPD group (*N* = 122)

	BPD group (*n* = 65)	Non-BPD group (*n* = 57)		
Variable	Mean or frequency (*SD* or %)	Mean or frequency (*SD* or %)	*t* or *χ²*	*p*
Age	34.05 (11.45)	34.75 (12.91)	0.32	0.749
Sex, female	49 (75.4%)	29 (50.9%)	7.910	0.005
GAF score	59.46 (10.64)	74.95 (12.62)	7.35	< 0.001
Mood disorders	25 (38%)	24 (42%)	0.17	0.682
Anxiety disorders	12 (18%)	7 (12.3%)	0.88	0.348
Obsessive-Compulsive and Related Disorders	2 (3%)	2 (3.5%)	0.02	0.894
Posttraumatic Stress Disorder	1 (1.5%)	1 (1.75%)	0.01	0.925
Adjustment disorder	1 (1.5%)	13 (22.8%)	13.52	< 0.001
Eating disorders	16 (24.6%)	2 (3.5%)	10.76	0.001
Substance-Related and Addictive Disorders	24 (37%)	2 (3.5%)	20.22	< 0.001
Somatic symptoms and related disorders	1 (1.5%)	1 (1.75%)	0.01	0.925
BPD	65 (100%)	0 (0%)	122	< 0.001
Other PDs	14 (21.5%)	7 (12.3%)	1.83	0.177

Note: Abbreviations: BPD, borderline personality disorder; PD, personality disorder. The Global Assessment of Functioning Scale [GAF; 69] rates the global functioning of the person and the impact of the symptoms on life on a score ranging from 1 to 100.

### Measures

#### The revised borderline follow-up interview (BFI-R)

The BFI-R [49] is a semi-structured interview measuring psychosocial functioning as well as treatment utilization during the two years prior to the interview. The instrument has previously been validated, exhibiting good to excellent levels of convergent validity, follow-up and longitudinal inter-rater reliability [15, 50, 51].

The information collected through the BFI-R [49] allowed us to derive the main variable of interest as well as various distinct aspects of functioning. Importantly, for the most part, items in the BFI-R are structured as close-ended questions, allowing participants to provide clear answers. Interviewers can ask follow-up questions for clarification if necessary. To exemplify this, an item from the BFI-R is: “How much distance or coolness has there been in this/these relationships?” with response options: 2 = no distance or coolness, 1 = some distance or coolness, 0 = substantial distance or coolness. The main variable of interest of the current paper has been operationalized in previous BPD literature [14] and is referred to as *overall functioning*. Good overall functioning is attained if the person (i) was able to perform consistently and competently in work or school over the past two years (including being a stay-at-home parent or carer), (ii) has at least one close and sustaining relationship with a partner of friend; the relationship needs to be characterized by regular contact without elements of abuse. In addition, we investigated educational, vocational, interpersonal, and social aspects of functioning that were, for the most part, based on the study conducted by Javaras et al. [20], with some minor changes. Specifically, the following aspects related to the education and vocational domains were investigated: completed years of education, educational/occupational status, educational/occupational functioning, and financial status. The following aspects were assessed regarding social and interpersonal domains: partnership status, partnership functioning, parenthood status, friendship status, friendship functioning, parents functioning, recreational status, and social isolation. Of note, no further interpretation nor coding of items was required to operationalize specific functioning categories; as such, given the nature of the information collected, the procedure employed to operationalize functioning categories, and their use in previous literature [20], their reliability was not specifically established in the present article. Detailed information on the operationalization of the specific categories of functioning is provided in Additional File 1.

#### The revised diagnostic interview for borderlines (DIB-R)

The DIB-R [38] is a semi-structured interview evaluating the two years prior to the interview with the aim of assessing BPD psychopathology. It provides a total score as well as four sub-scores for the following domains of BPD symptomatology: affect, cognition, impulse action patterns, and interpersonal relationships.

The interview has 127 items, which include summary statements and section scores. More specifically, items are grouped in 22 summary statements. Each item and summary statement is rated as 2 (Yes), 1 (Probable) and 0 (No). The total scores for each domain are calculated by summing the scores of their respective summary statements. Subsequently, each domain total score is converted into a scaled section score based on the instructions provided in the interview. The DIB-R [38] total score ranges from 0 to 10 and a score of 8 or more is indicative of the presence of BPD. The French version of the DIB-R showed good psychometric properties [52]. In addition, as presented in the French validation article, results of t-tests indicate that the two groups statistically differ on the general score as well as on all sub-scores of the DIB-R (DIB-R total score: *t*= -10.59, *p* < 0.001, affect domain: *t*=-3.71, *p* < 0.001, cognition domain: *t*=-6.62, *p* < 0.001, impulse action patterns domain: *t*= -8.32, *p* < 0.001, interpersonal relationships domain: *t*= -9.35, *p* < 0.001).

### Procedure

The research was approved by the competent ethics committee (number 2016–02235). The results presented in the current paper are part of an ongoing longitudinal study conducted at a French-speaking University Hospital. During assessment, two semi-structured interviews are administered in order to assess BPD psychopathology as well as social and vocational functioning; additionally, participants are asked to fill out a number of self-report questionnaires. Interviews, which were generally videotaped, were conducted by one PhD student and one research assistant. To evaluate participants, the two researchers underwent comprehensive training in conducting semi-structured interviews. This training encompassed the observation and assessment of multiple interviews conducted by experienced researchers. Additionally, they received guidance from the primary developer of the scales, offering an opportunity to address any uncertainties or concerns.

### Statistical analyses

All domains of functioning assessed were categorial variables except for the years of education and social isolation that were considered as continuous. For categorical variables, we defined one reference category indicating the least desirable category; the reference category is also reported in the tables depicting the results.

In order to test the associations between (i) BPD status; (ii) intensity of BPD symptom domains (affective, cognitive, impulse action pattern, interpersonal) assessed with the DIB-R and different aspects of functioning we ran separate multiple regression models. More specifically, we ran logistic models when the outcome variables had two categories, multinomial models when the outcome variable had more than two categories and linear model when it was continuous (i.e. years of education and social isolation).

In addition for the functioning variables: educational/occupational functioning, partnership functioning, friendship functioning, parents functioning (exclusively when testing the association with the intensity of BPD symptom domains) we employed Poisson log-linear models [53] to estimate the parameters of the multinomial logistic regressions. This was done to reduce the bias and overcome the problem of instability in the model fits. Furthermore, for each model, Omnibus Wald tests were conducted to assess the overall effect of (i) BPD status, and (ii) intensity of BPD symptom domains. From the multinomial logistic regressions, Relative Risks (RR) were reported for the impact of both (i) BPD status, and (ii) intensity of BPD symptom domains on each aspect of functioning in respect to the least desirable category (reference category). For logistic and linear regressions, Odds ratios and β estimates were reported respectively.

All models were adjusted for the following covariates: age, gender and number of months during covid-19 pandemic (i.e. months of covid-19 pandemic during the 24 previous months). More precisely, we defined the covid-19 pandemic in the country where the study took place as the period starting on March 16th, 2020, when the first lockdown was announced, and ending on April 1st, 2022, when all remaining measures were lifted. This decision was made taking into account the significant impact the pandemic could potentially have on both professional and social aspects of life.

All analyses were performed using the R environment for statistical computing version 4.1.0 [54] and 0.05 was taken as significance level for the reported statistical tests.

## Results

### Good overall psychosocial functioning

**Fig. 1:**
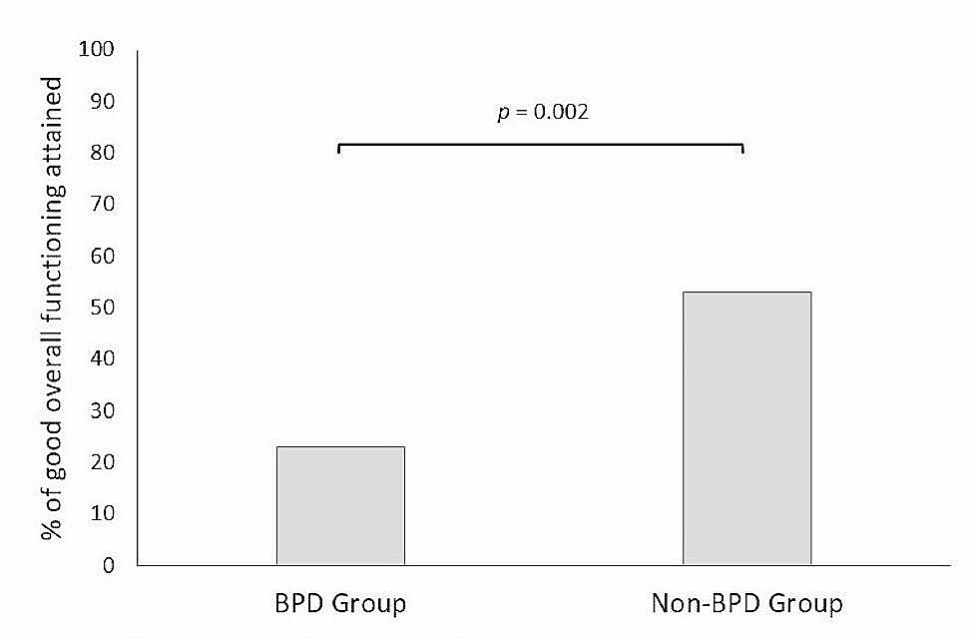
Rates of participants in the BPD and non-BPD groups who attained good overall functioning. *Note*: A logistic regression was run to test the association between BPD status and good overall functioning. The model was adjusted for age, gender and number of months during covid-19 pandemic. Results indicate that BPD participants are statistically less likely to achieve good psychosocial functioning compared to non-BPD participants (OR = 0.29, 95% CI [0.13, 0.63], *p* = 0.002). In the present sample, 23% of participants in the BPD group and 53% in the non-BPD group attained good psychosocial functioning

The percentages of participants who attained good overall psychosocial functioning in each group are displayed in Fig. 1. In the BPD group, 23% of participants met the criteria indicating good overall functioning, whereas in the non-BPD group, this percentage was 53%. Results of the logistic regression indicate that BPD participants are statistically less likely to achieve good psychosocial functioning compared to non-BPD participants (OR = 0.29, 95% CI [0.13, 0.63], *p* = 0.002).

When considering the entire sample, the four sub-scores of the DIB-R were not significantly associated with the probability of attaining good psychosocial functioning (affective domain: OR = 1, 95% CI [0.81, 1.23 ], *p* = 0.990; cognition domain: OR = 0.85, 95% CI [0.67, 1.09], *p* = 0.198; impulse action patterns domain: OR = 0.87, 95% CI [0.71, 1.06], *p* = 0.180; interpersonal domain: OR = 0.92, 95% CI [0.79, 1.07], *p* = 0.265).

### Educational and vocational domains of functioning

The results of the educational and vocational functioning domains that compare groups with and without BPD are presented in Table 2. As indicated by the Omnibus Wald test, there was a statistically significant difference between the BPD and non-BPD groups across all functioning domains. More specifically, being in the BPD group was associated with lower number of completed years of education. Regarding educational/occupational status, BPD participants were less likely to work consistently and on a full-time basis for a salary compared to participants in the non-BPD group. The results for educational/occupational functioning indicate an overall statistically significant difference between the two groups but the difference between specific categories could not be detected in the model. However, as indicated by risk ratios and estimated category proportions, BPD participants seem less likely to be working at a high or satisfactory level, they also have a higher likelihood of not working at all; this observation might be attributed to the limited representation in the reference category for the non-BPD group, which included only one participant. Lastly, the results for financial status indicate that participants in the BPD group are less likely to be fully or partially independent compared to the non-BPD group, as, in fact, a majority (55%) of BPD participants is fully dependent.

**Table 2:** Educational and vocational domains of functioning for the BPD (*N* = 65) vs. NON-BPD (*N* = 57) groups

Educational and vocational domain outcomes	Omnibus Wald Test	Risk ratios/β			Estimated category proportions (or mean)	
	χ², df (*p* value)	Estimate	95% CI	*p*-value	BPD, *n* (%) or M (SD)	NON-BPD, *n* (%) or M (SD)
Years of education^1^	5.95, 1 (< 0.01)	β = -1.46	-2.65 – -0.26	0.017	13.06 (3.36)	14.79 (3.09)
Educational/Occupational status^2^						
Consistent work – paid		0.13	0.04–0.41	0.001	10 (16%)	30 (53%)
Consistent work- student/carer		0.85	0.17–4.11	0.837	10 (16%)	5 (8.8%)
Some work/study		0.56	0.17–1.83	0.332	23 (36%)	15 (26%)
No work/Study (R)		–	–	–	21 (33%)	7 (12%)
Overall	17.55, 3 (< 0.001)					
Educational/Occupational functioning^3^						
High or satisfactory level		0.34	0.05–2.27	0.266	20 (34%)	36 (65%)
Mild impairment		0.76	0.10–5.57	0.791	14 (24%)	12 (22%)
No work at all		2.11	0.27–16.51	0.478	22 (37%)	6 (11%)
Moderate/severe impairment (R)		–	–		3 (5.1%)	1 (1.8%)
Overall	13.21, 3 (< 0.001)					
Financial status^2^						
Fully independent		0.17	0.05–0.53	0.003	12 (26%)	22 (52%)
Partially dependent		0.18	0.05–0.65	0.009	9 (19%)	13 (31%)
Fully dependent, illness/disability (R)		–	–	–	26 (55%)	7 (17%)
Overall	12.59, 2 (< 0.001)					

Note: All models are adjusted for age, gender and number of months during covid-19 pandemic.

^1^ Linear model

^2^ Multinomial logistic regression

^3^ Poisson log-linear model

The results of the associations between the intensity of DIB-R symptom domains and the educational and vocational domains of functioning for the total sample are presented in Table 3. The only statistically significant associations between the intensity of BPD symptom domains assessed using the DIB-R and educational/vocational domains of functioning are the following: the intensity of the DIB-R cognitive domain was associated with lower chances of consistently working for a salary and higher chances of not working at all; a higher DIB-R interpersonal score was associated with lower chances of being partially financially dependent.

**Table 3:** Associations between the intensity of DIB-R symptom domains and educational and occupational domains of functioning (*N* = 122)

	Risk ratios /β												Omnibus Wald test
Outcome	DIB-*R* Affective			DIB-*R* Cognitive			DIB-*R* Impulsiveness			DIB-*R* Interpersonal			Overall
	Est.	95% CI	*p*	Est.	95% CI	*p*	Est.	95% CI	*p*	Est.	95% CI	*p*	χ², df (p value)
**Years of education** ^**1**^	β = 0.04	-0.26–0.35	0.774	β = -0.32	-0.68–0.04	0.081	β = -0.06	-0.35–0.22	0.652	β = -0.05	-0.26–0.16	0.622	9.33, 4 (0.05)
**Educ./Occupational status** ^**2**^													27.47, 12 (0.01)
Consistent work – paid	1.10	0.80–1.50	0.558	0.67	0.47–0.97	0.033	0.78	0.59–1.05	0.105	0.93	0.76–1.14	0.477	
Consistent work- student/carer	0.83	0.55–1.48	0.355	0.90	0.55–1.48	0.666	0.88	0.62–1.25	0.464	1.01	0.80–1.50	0.558	
Some work/study	1.03	0.74–1.42	0.873	0.75	0.52–1.10	0.137	0.96	0.73–1.25	0.747	1.01	0.83–1.22	0.944	
No work/Study (R)	-				-			-			-		
**Educ./Occupational functioning** ^**3**^													33.97, 12 (< 0.001)
High or satisfactory level	0.29	0.07–1.20	0.088	1.35	0.76–2.39	0.311	1.09	0.70–1.69	0.698	0.84	0.61–1.14	0.257	
Mild impairment	0.30	0.07–1.26	0.100	1.60	0.87–2.93	0.132	1.09	0.69–1.72	0.710	0.89	0.64–1.23	0.476	
No work at all	0.29	0.07–1.19	0.085	2.02	1.08–3.78	0.029	1.28	0.81–2.02	0.296	0.90	0.65–1.24	0.507	
Moderate/severe impairment (R)													
**Financial status** ^**2**^													22.4, 8 (< 0.001)
Fully independent	1.12	0.80–1.57	0.506	0.85	0.60–1.22	0.385	0.89	0.66–1.20	0.436	0.82	0.67–1.02	0.078	
Partially dependent	1.18	0.82–1.70	0.360	0.88	0.60–1.30	0.520	0.84	0.62–1.15	0.273	0.78	0.62–0.99	0.043	
Fully dependent, illness/disability (R)													

Note: All models are adjusted for age, gender and number of months during covid-19 pandemic.

^1^ Linear model

^2^ Multinomial logistic regression

^3^ Poisson log-linear model

### Interpersonal and social domains of functioning

The results of the interpersonal and social functioning domains that compare groups with and without BPD are presented in Table 4. As indicated by the Omnibus Wald test, there was an overall statistically significant difference between the BPD and non-BPD groups in partnership status and partnership functioning, friendship functioning and parents functioning. More specifically, BPD participants were statistically less likely to be in a cohabiting relationship, in addition, the estimated category proportions shows that 55% of participants in the BPD group was not in a romantic relationship compared to 38% in the non-BPD group. The comparison between the two groups also revealed a statistically significant difference in overall friendship functioning, however, no significant differences were observed in specific aspects of functioning; when examining estimated category proportions alone, we observe that non-BPD participants show 45% of very good/good friendships, whereas the BPD group shows a lower rate at 28%, still, this difference is not statistically significant. In respect to parents functioning, participants in the BPD group exhibited lower chances of having very good or good relationships with both parents and lower chances of having one very good/good relationship with one parent and a less than good relationship with the other.

The results of the associations between the intensity of DIB-R symptom domains and the interpersonal and social domains for the total sample are presented in Table 5. The results indicate that higher scores in the affective domain are associated with lower chances of being in a cohabiting relationship and higher chances of being socially isolated; higher scores in the cognitive domain are associated with higher chances of being socially isolated; higher scores in the interpersonal relationship domain are associated with lower chances of having one positive relationship with a parent and the other one less than good, as well as higher chances of being socially isolated.

**Table 4:** Interpersonal and social domains of functioning for the BPD (*N*=65) vs NON-BPD (*N*=57) groups

Interpersonal and social domains outcomes	Omnibus Wald Test	Risk ratios/OR/ β			Estimated category proportions	
	χ², df (*p* value)	Estimate	95% CI	*p*-value	BPD, *n* (%) or M (SD)	NON-BPD, *n* (%) or M (SD)
Partnership status^1^						
Cohabiting		0.36	0.14–0.94	0.036	14 (22%)	20 (36%)
Steady but not cohabiting relationship		0.45	0.17–1.19	0.105	15 (23%)	15 (27%)
Not in a steady relationship (R)		–	–	–	35 (55%)	21 (38%)
Overall	5.49, 2 (0.06)					
Partnership functioning^2^						
Very good/good relationship		0.14	0.01– 1.89	0.140	18 (28%)	25 (45%)
Fair relationship		0.20	0.01– 3.00	0.246	8 (12%)	9 (16%)
No relationship		0.37	0.03–4.88	0.450	35 (55%)	22 (39%)
Poor/Very poor relationship (R)					3 (4.7%)	0 (0%)
Overall	7.12, 3 (0.07)					
Parenthood status^3^						
Has children		OR = 0.49	0.18–1.33	0.160	16 (25%)	20 (35%)
No children (R)		–	–	–	49 (75%)	37 (65%)
Overall	2.04, 1 (0.15)					
Friendship status^1^						
Five or more friends		0.45	0.14–1.52	0.199	18 (28%)	18 (32%)
Two to four friends		0.41	0.14 − 1.24	0.114	31 (48%)	31 (55%)
Zero or one friend (R)		–	–	–	15 (23%)	7 (12%)
Overall	2.76, 2 (0.25)					
Friendship functioning^2^						
Very good/good relationship(s)		1.38	0.20–9.62	0.745	39 (61%)	46 (82%)
Fair relationship(s)		6.02	0.68–53.04	0.106	16 (25%)	4 (7.1%)
Poor/Very poor relationship(s) (R)		–	–	–	1 (1.6%)	2 (3.6%)
No friends		3.03	0.33–27.70	0.327	8 (12%)	4 (7.1%)
Overall	9.23, 3 (0.03)					
Parents functioning^1^						
All relationships good/very good		0.08	0.01–0.45	0.005	12 (19%)	22 (39%)
One good/very, other less than good		0.15	0.02–0.98	0.048	14 (22%)	13 (23%)
One fair, other fair or worse		0.22	0.04–1.26	0.087	23 (36%)	17 (30%)
All relationships poor, very poor (R)		–	–	–	12 (19%)	2 (3.6%)
No parents alive		0.19	0.01–2.64	0.214	3 (4.7%)	2 (3.6%)
Overall	11.98, 4 (0.02)					
Recreational status^1^						
At least weekly participation		0.90	0.24–3.32	0.868	45 (70%)	46 (82%)
Some participation, but less than weekly		1.88	0.36–9.76	0.449	13 (20%)	5 (8.9%)
No participation (R)		–	–	–	6 (9.4%)	5 (8.9%)
Overall	1.66, 2 (0.44)					
Social isolation (% of time spent alone)^4^	2.08, 1 (0.15)	β = 7.19	-2.85–17.23	0.159	54.89 (27.65)	47.31 (25.47)

Note: All models are adjusted for age, gender and number of months during covid-19 pandemic.

^1^ Multinomial logistic regression

^2^ Poisson log-linear model

^3^Logistic regression

^4^ Linear model

**Table 5:** Associations between the intensity of DIB-R symptom domains and interpersonal and social domains of functioning (*N*=122)

Outcome	Risk ratios/Odds ratio/β												Omnibus Wald test
	DIB-*R* Affective			DIB-*R* Cognitive			DIB-*R* Impulsiveness			DIB-*R* Interpersonal			Overall
	Est.	95% CI	*p*	Est.	95% CI	*p*	Est.	95% CI	*p*	Est.	95% CI	*p*	χ², df (*p* value)
**Partnership status** ^**1**^													13.34, 8 (0.10)
Cohabiting	0.73	0.57–0.94	0.015	1.01	0.75–1.34	0.964	0.96	0.77–1.20	0.725	1.05	0.89–1.25	0.563	
Steady but not cohabiting relationship	0.84	0.64–1.10	0.207	0.78	0.57–1.07	0.123	1.15	0.97–1.38	0.111	1.15	0.97–1.38	0.111	
Not in a steady relationship (R)													
**Partnership functioning** ^**2**^													18.02, 12 (0.12)
Very good/good relationship	1.03	0.69–1.53	0.894	1.39	0.76–2.55	0.288	0.70	0.45–1.10	0.123	1.05	0.73–1.50	0.805	
Fair relationship	1.36	0.84–2.18	0.209	1.19	0.63–2.27	0.595	0.83	0.51–1.34	0.444	1.04	0.71–1.51	0.851	
No relationship	1.41	0.92–2.14	0.114	1.46	0.80–2.67	0.214	0.76	0.49–1.19	0.236	0.95	0.67–1.35	0.762	
Poor/Very poor relationship (R)													
**Parenthood status** ^**3**^													2.37, 4 (0.67)
Has children	OR = 1.05	0.80 − 1.38	0.712	OR = 0.84	0.62–1.14	0.270	OR = 1.05	0.82–1.34	0.710	OR = 1.09	0.91–1.31	0.351	
No children (R)													
**Friendship status** ^**1**^													12.88, 8 (0.12)
Five or more friends	0.97	0.70–1.35	0.872	0.84	0.57–1.23	0.361	0.97	0.73–1.29	0.845	0.91	0.74–1.12	0.377	
Two to four friends	1.04	0.77–1.40	0.813	1.10	0.78–1.55	0.599	0.80	0.62–1.04	0.092	0.95	0.78–1.14	0.555	
Zero or one friend (R)													
**Friendship functioning** ^**2**^													25.72, 12 (0.01)
Very good/good relationship(s)	0.59	0.24–1.41	0.231	0.93	0.52–1.66	0.806	1.19	0.76–1.88	0.450	0.87	0.64–1.18	0.357	
Fair relationship(s)	0.53	0.21–1.32	0.173	1.02	0.53–1.94	0.961	1.47	0.90–2.42	0.127	0.97	0.69–1.36	0.843	
Poor/Very poor relationship(s) (R)													
No friends	0.45	0.18–1.12	0.087	1.44	0.70–2.96	0.323	1.49	0.89–2.49	0.126	0.86	0.60–1.22	0.390	
**Parents functioning** ^**2**^													32.34, 16 (0.01)
All relationships good/very good	0.91	0.61–1.37	0.664	0.83	0.53–1.29	0.401	0.93	0.66–1.30	0.661	0.90	0.68–1.18	0.446	
One good/very, other less than good	1.07	0.69–1.65	0.757	0.97	0.60–1.58	0.905	1.13	0.80–1.59	0.490	0.68	0.51–0.91	0.009	
One fair, other fair or worse	1.12	0.74–1.71	0.596	0.79	0.51–1.24	0.304	1.11	0.80–1.53	0.529	0.84	0.65–1.09	0.180	
All relationships poor, very poor (R)													
No parents alive	2.71	0.79–9.33	0.114	0.64	0.33–1.23	0.179	1.04	0.65–1.65	0.876	0.94	0.66–1.32	0.709	
**Recreational status** ^**1**^													7.41, 8 (0.49)
At least weekly participation	0.96	0.69–1.33	0.800	0.94	0.62–1.41	0.750	0.75	0.55–1.03	0.073	1.22	0.96– 1.54	0.099	
Some participation, but less than weekly	0.90	0.60–1.34	0.586	1.14	0.69–1.90	0.601	0.87	0.60–1.25	0.454	1.05	0.80–1.37	0.733	
No participation (R)													
**Social isolation**^**4**^ (% of time spent alone)	β = 3.03	0.60–5.46	0.015	β = 3.62	0.77–6.47	0.013	β = 1.56	-0.63–3.74	0.160	β = -2.35	-3.98–-0.73	0.005	23.05, 4 (< 0.001)

Note: All models are adjusted for age, gender and number of months during covid-19 pandemic.

^1^ Multinomial logistic regression

^2^ Poisson log-linear model

^3^Logistic regression

^4^Linear model

## Discussion

The main goal of the present study was to compare psychosocial functioning of a group with BPD and a clinical comparison group of individuals without BPD. Specifically, the two groups were compared in terms of overall functioning, and specific aspects of functioning within educational/vocational, interpersonal, and social domains. We hypothesized that the BPD group would exhibit poorer functioning. An additional goal of the study was to explore associations between the intensity of BPD symptom sectors and the afore-mentioned aspects of functioning.

Regarding the first aim of the study, the analyses revealed a reduced likelihood for the BPD group to fulfill criteria for good overall psychosocial functioning; for clarity, this implied that the person was able to work, study or care consistently and have at least one close and sustaining relationship with a close friend or partner. In our sample, only 23% of participants in the BPD group met the criteria defining good psychosocial functioning, whereas this percentage was 53% in the comparison group. More specifically, among participants with BPD who did not fulfill criteria for good psychosocial functioning, 26.5% did not meet any of the necessary criteria, approximately 10% only met the education/occupation criterion, and about 63% had at least one good and sustainable relationship but was not able to work, study or care consistently. The results of the present study are in line, among others, with the findings of two important longitudinal studies on the course of BPD. The McLean Study of Adult Development [15] found that good overall psychosocial functioning at baseline was attained by only 25.9% of participants with BPD; this percentage increased to 50% at the 10-year follow-up and to 60% at the 16 and 20 years follow-up [11]. Similarly, the Collaborative Longitudinal Personality Disorders study [55], observed that at baseline 19% of BPD participants worked full time and 23% were married or in a cohabiting relationship. When moving from this general definition of functioning to the specific aspects of functioning, as hypothesized, we observed that the main differences between the two groups were found in the educational/vocational domain, where BPD participants exhibited worse functioning. These differences implied lower completed years of education, a lower likelihood of having consistent and satisfactory employment, as well as higher odds of being financially dependent. These results are consistent with previous studies observing that the occupational domain seemed particularly impaired in BPD [e.g., 6, 31, 55]. In light of the findings presented above, which highlight differences observed in the BPD and non-BPD samples, it is essential to consider additional demographic factors that may influence these outcomes. Notably, the gender composition of our samples merits particular attention. The BPD sample, in particular, exhibited a significant majority of women (75.4%), unlike the non-BPD sample. Given the well-documented gender discrimination in the occupational domain [56, 57], which negatively affects women, as well as the cumulative impact of mental health challenges on employment outcomes [58], the gender composition of our samples is particularly relevant. This demographic characteristic highlights the importance of acknowledging differences in the composition of our sample.

When it comes to interpersonal relationships, the main differences observed between the BPD and non-BPD groups were the following: in the BPD group, cohabiting relationships were less frequent and, more generally, the rate of BPD participants involved in a romantic relationship was lower than that of the comparison group (still, this difference was not statistically significant); in addition, BPD participants were less likely to have good relationships with their parents. A number of previous studies showed that individuals with a BPD diagnosis reported higher chances of being divorced, separated or never married [e.g., 18, 22] as well as having dysfunctional romantic relationships [59]. Even though in our sample more non-BPD participants were in a relationship compared to BPD participants, a total of 45% of individuals in the BPD group was in a romantic relationship, which, interestingly, is comparable to the rate observed by Zeitler et al. [10] in a sample of symptomatically remitted BPD participants.

In addition to areas of impairment, it is equally necessary to highlight domains where individuals exhibit good functioning, as these can act as valuable resources. For instance, current results suggest that relationships with friends and recreational activities can play an important role in fostering well-being and quality of life. In fact, 88% of participants in the BPD group (compared to 93% in the non-BPD group) had at least one close friend and, apart from one exception, the quality of these relationships was always fair to good. Moreover, 70% of participants in the BPD group reported at least weekly participation in a recreational activity, these included community activities, involvement in organizations or clubs, participation in religious or spiritual activities, engagement in hobbies and sports. In comparison, this percentage rose to 82% in the non-BPD group, which did not statistically differ from the BPD group.

The present results, which identify impaired domains of functioning as well as resources, contribute to existing evidence and help in pinpointing areas that require attention and targeting in treatment, as well as recognizing domains that can act as facilitators. This is particularly relevant especially when considering findings indicating that patients sometimes feel that treatment goals do not correspond to their personal goals [60]. In addition, it is crucial to not only prioritize psychosocial functioning in therapy, but also in treatment research by conducting high-quality studies [41, 42]. Undoubtedly, quantitative research should be complemented by studies taking more explicitly into account personal definitions of recovery from a subjective perspective. For instance, Zeitler et al. [10] showed that perceived social support predicted reported life satisfaction among BPD patients. However, it is reasonable to assume that, despite reporting a certain number of close relationships, a feeling of subjective loneliness or social isolation could still persist and negatively impact quality of life. As an illustration, Liebke et al. [61] noted that social isolation and functioning alone did not adequately account for the heightened feeling of loneliness reported by BPD patients. It is encouraging to see that a growing effort is being made in order to take into account personal definitions and dimensions of recovery as reported by clients [60, 62–64], and we can assume that research might greatly benefit from the addition of qualitative studies to more objective and quantitative investigations [65].

The secondary aim of the current study was to assess the relationships between intensity of BPD symptom sectors and functioning for the total sample. Investigating the intensity of BPD symptoms also holds significance for the dimensional perspective of personality disorders, particularly in identifying specific aspects that contribute to their severity [66, 67]. As several studies showed that BPD symptoms predict functioning [5, 20, 40], we anticipated observing a more systematic association between intensity of symptom sectors and functioning than what the current results revealed. Specifically, contrary to previous research [20, 39, 40, 46] no association was observed between the intensity of BPD symptom sectors and good overall psychosocial functioning. Therefore, our initial hypothesis on a significant association between the impulse action domain symptom sector and overall functioning was not confirmed. A potential reason for this lack of association may be that the present hypothesis was based on previous literature that focused exclusively on participants with BPD. In contrast, for the second aim of the present study, we analyzed the entire sample, which included participants in the non-BPD group who might exhibit low scores of BPD symptoms. However, we were able to identify a number of associations. Our results revealed that high scores in the affect, cognition and interpersonal relationships domains all contributed to a higher degree of time spent alone. In addition, it appears that the cognition domain, which comprises odd thinking, unusual perceptual experiences, and non-delusional paranoid experiences, is mostly related to impairment in the educational/vocational domain, while the affective and interpersonal relationships domains are mostly related to impairment in relationships with romantic partners and parents. These results should be interpreted with caution and warrant a reflection on possible explanations for the lack of associations. First, it might be plausible that some confounding variables that have not been taken into account in the present study, such as socioeconomic status, level of education, or comorbidities, play a role in the observed phenomenon. Second, considering that for the second aim of the study the sub-groups were not considered separately, it might be that the variability of BPD symptom sectors’ scores was too low in the non-BPD group; in addition, it is important to mention the heterogeneity of the non-BPD group. Third, there may be some methodological implications of employing a semi-structured interview investigating the two years prior to the interview: even though information was gathered using a validated interview in a research context, the fact that participants had to recall information relating to a relatively long period of time might limit the ability to capture fluctuations in symptoms in a fine-grained manner. It is possible that important information could be lost, and employing other methodologies, for instance ecological momentary assessment, might facilitate a better understanding of the complexities of daily functioning and symptoms.

There are a number of limitations of the present study that need to be acknowledged. First, the sample sizes of both groups included can be considered as small. However, especially in respect to the first aim of the study, the results go in the direction of previous research, which suggests a certain degree of reliability of current intepretations. Second, in this study, we analyzed data collected concurrently at a single time point; in order to establish predictors over time, cross-lagged analyses should be conducted. Third, the current analyses were not adjusted for comorbidities, however information on comorbid disorders for the two groups is reported in Table 1. Of note, the rate of some comorbid disorders, such as PTSD, is lower compared to what is typically reported in the literature. This can be explained by the fact that only BPD was systematically assessed, whereas other comorbid disorders relied on clinicians’ evaluations in a naturalistic context within a specialized clinic focused on personality disorders. This approach may have contributed to the underreporting of comorbid conditions such as PTSD, and a primary focus on diagnoses of personality disorders. However, this does not exclude the possibility of a high presence of trauma within our study sample. Fourth, the non-BPD comparison group was hetergenous in respect to diagnosis. Fifth, the definitions of functioning are mainly based on objective criteria and often fail to take into account the personal view of clients and what they find satisfying or, on the contrary, unsatisfying. Furthermore, more validity data would be important for the definition of good overall psychosocial functioning. Also, as previously described, no reliability was established for the specific functioning categories; however our procedure is in accordance with previous literature [20]. Sixth, as already stated in the discussion, when retrospectively reporting on the two previous years, there might be recall biases influencing the participants’ answers. Seventh, we explored four symptom domains which included a variety of symptoms. Future research may benefit from a more in-depth exploration of specific symptoms that might exert a unique influence, such as chronic emptiness or identity issues. Eighth, in the present study the BPD group exhibited significantly lower scores on the GAF compared to the non-BPD group, suggesting a higher level of symptom severity and impairment. In order to provide a more comprehensive understanding of the findings, future research could benefit from including an additional comparison group. For instance, incorporating a group with chronic schizophrenia or bipolar disorder, matched for GAF scores with the BPD group, would allow for a more nuanced analysis of functional outcomes across different psychiatric conditions. Lastly, evidence has established that the breadth of personality disorders as well as traits are related to dysfunction [e.g., 40, 67, 68], however they were not assessed in the current study.

## Conclusions

In conclusion, it is crucial to pursue research efforts focusing on psychosocial functioning which will allow us to better characterize functional impairment, identify potential strengths and resources, tailor treatment approaches, all the while integrating clients’ personal perspectives in what defines good functioning. In sum, the focus should be on “extending beyond only the individual and their symptoms and focusing on elements that foster a life worth living” [65, p.151].

### Electronic supplementary material

Below is the link to the electronic supplementary material.

Supplementary Material 1

## Data Availability

The data that support the findings of this study are available from the corresponding author upon reasonable request.
