# Supplementary material for: Symptom domains and psychosocial functioning in borderline personality disorder
Source: Borderline Personal Disord Emot Dysregul. 2024 Jun 5;11:10. doi: 10.1186/s40479-024-00255-2 (PMC11151627; doi:10.1186/s40479-024-00255-2)
Supplement: Supplementary file 1 — Supplementary Material 1 [file 40479_2024_255_MOESM1_ESM.docx]

**Supplemental material**

*Additional file 1*

Operationalization of functional outcomes based on the information derived from the BFI-R.

| **Variable** | **Outcome** | **Definition** |
| --- | --- | --- |
| Main outcome: Overall functioning | Good | At least one emotionally sustaining relationship with a close friend or romantic partner, the relationship is close with no or little conflict AND ability to work/study/care full-time in a consistent, competent way (All 24 months in the past two years) |
|  | Bad | Above definition not met |
| Years of education  *(Educational/vocational domain)* |  | Total number of completed years of schooling |
| Educational/Occupational status  *(Educational/vocational domain)* | Consistent work for salary | The person is working almost all the time for salary (All 24 months in the past two years.) |
|  | Consistent work as student or carer | The person is working almost all the time as student or as a houseperson/carer (All 24 months in the past two years) |
|  | Some work or study | The person is working for salary or as a student, houseperson/carer some of the time (in the last 24 months, for a duration ranging from 1 to 23 months) |
|  | No work/study (R) | The person has not worked during the past 24 months |
| Educational/occupational functioning  *(Educational/vocational domain)* | High or satisfactory level | During the last 24 months, the person has maintained a work history that is at least steady. |
|  | Mild impairment | During the last 24 months, the person had a work history that is somewhat troubled |
|  | Moderate/severe impairment (R) | During the last 24 months, the person had a work history that is very troubled |
|  | No work at all (0) | The person did not work at all in the past 24 months |
| Financial status  *(Educational/vocational domain)* | Fully independent | The person is financially independent (or needs occasional support) or is relying on family support because of student/houseperson/ carer status |
|  | Partially dependent | The person requires financial support more frequently than occasionally, but is not fully dependent. The financial support is not explained by student/houseperson/ carer status |
|  | Fully dependent because of illness or disability (R) | The person is fully financially dependent on family or government due to illness |
| Partnership status  *(Interpersonal/social domain)* | Cohabiting | The person is living with a spouse or steady partner |
|  | Steady but not cohabiting relationship | The person is in a steady relationship, but is not cohabiting with the partner |
|  | Not in a steady relationship (R) | The person is not currently in a steady relationship |
| Partner functioning  *(Interpersonal/social domain)* | Very good/good relationship | The current relationship with a partner is considered as close with no or little conflict |
|  | Fair relationship | The current relationship is considered as close with substantial conflict, somewhat distant, or somewhat stormy |
|  | Poor/very poor relationship (R) | The current relationship is considered as very distant, very stormy, abusive, or there is no contact |
|  | No relationship | The person is not currently in a relationship |
| Parenthood status  *(Interpersonal/social domain)* | Has children | The person has one or more biological, adopted or step-children |
|  | No children (R) | The person has no children |
| Friendship status  *(Interpersonal/social domain)* | Five or more friends | The person has five or more friends |
|  | Two to four friends | The person has two to four friends |
|  | Zero to one friend (R) | The person has one friend or no friends |
| Friends functioning  *(Interpersonal/social domain)* | Very good/good relationship | During the last 24 months, the person had friendships that are close and with little or no conflict |
|  | Fair relationship | During the last 24 months, the person had friendships that are close with substantial conflict, somewhat distant, or somewhat stormy |
|  | Poor/very poor relationship (R) | During the last 24 months, the person had friendships that are very distant, very stormy, abusive, or there is no contact |
|  | No friends (O) | The person does not have any close friends |
| Parents functioning  *(Interpersonal/social domain)* | All relationships good/very good | All parental relationships are positive, ranging from good to very good, characterized by closeness and minimal conflict |
|  | One good/very good, other less than good | One parental relationship is characterized as good to very good, with closeness and minimal conflict, while the other is not at the same positive level |
|  | One fair, other fair or worse | One parental relationship is rated as fair, (close with substantial conflict/ somewhat distant/ somewhat stormy), if the other relationship exists, it is characterized as fair/poor/very poor |
|  | All relationships poor/very poor (R) | All existing parental relationships are poor/very poor (very distant, very stormy, abusive, no contact) |
|  | No parents alive (O) | No parents alive |
| Recreational status  *(Interpersonal/social domain)* | At least weekly participation | The person participates at least weekly in community activities/organizations/clubs/religious or spiritual activities/hobbies/sports |
|  | Some participation, but less than weekly | The person participates less than weekly in community activities/organizations/clubs/religious or spiritual activities/hobbies/sports |
|  | No participation (R) | The person never participates in community activities/organizations/clubs/religious or spiritual activities/hobbies/sports |
| Social Isolation  *(Interpersonal/social domain)* | % of time spent alone | Estimation of the percentage of free time that the person spends alone |
| *Note:* The operationalization of good overall functioning is based on the The McLean Study of Adult Development (MSAD; Zanarini et al., 2005), that of specific functional outcomes is highly based on Javaras et al. (2017). | | |
